# Bombesin receptor-targeted liposomes for enhanced delivery to lung cancer cells

**DOI:** 10.3762/bjnano.10.246

**Published:** 2019-12-19

**Authors:** Mohammad J Akbar, Pâmela C Lukasewicz Ferreira, Melania Giorgetti, Leanne Stokes, Christopher J Morris

**Affiliations:** 1 School of Pharmacy, University of East Anglia, Norwich, UK

**Keywords:** bombesin, GRPR, liposome, lung cancer, targeting

## Abstract

**Background:** Gastrin-releasing peptide is a member of the bombesin family of peptides. Its cognate receptor, gastrin releasing peptide receptor (GRPR), is widely expressed in cancers of the lung, pancreas and ovaries. Gastrin releasing peptide (GRP) is an autocrine growth factor in small cell lung cancer, which has very poor patient outcomes. High affinity antagonist peptides have been developed for in vivo cancer imaging. In this report we decorated pegylated liposomes with a GRPR antagonist peptide and studied its interaction with, and accumulation within, lung cancer cells.

**Results:** An N-terminally cysteine modified GRPR antagonist (termed cystabn) was synthesised and shown to inhibit cell growth in vitro. Cystabn was used to prepare a targeted 1,2-distearoyl-*sn*-glycero-3-phosphoethanolamine-*N*-[amino(polyethylene glycol)-2000] (DSPE-PEG2000) lipid conjugate that was formulated into liposomes. The liposomes displayed desirable colloidal properties and good stability under storage conditions. Flow cytometric and microscopic studies showed that fluorescently labelled cystabn-decorated liposomes accumulated more extensively in GRPR over-expressing cells than matched liposomes that contained no cystabn targeting motif.

**Conclusion:** The use of GRPR antagonistic peptides for nanoparticle targeting has potential for enhancing drug accumulation in resistant cancer cells.

## Introduction

Small-cell lung cancer (SCLC) accounts for approximately one in five lung cancer diagnoses. In spite of global efforts to reduce tobacco smoking in recent decades, the incidence of smoking-associated cancers such as SCLC remains high with more than 142,000 deaths from lung cancer estimated in the United States for 2019 [1]. Approximately 80% of the world's 1.1 billion smokers live in low- and middle-income countries, where access to state of the art healthcare and diagnostic technologies is limited [2]. Alarmingly, the average life expectancy of an untreated SCLC patient is less than four months due to the high likelihood of diagnosis at the metastatic stage. With intensive chemo- and radiotherapy the median survival extends to only 14–18 months meaning new therapeutic approaches for targeted drug delivery to SCLC are desperately needed.

SCLC belongs to a class known as neuroendocrine tumours in which malignant cells secrete hormones and growth factors – a trait inherited from the neuroendocrine cells of the bronchial epithelium that are transformed and give rise to the early SCLC tumours. Of the hormones known to be secreted by SCLC cells, gastrin-releasing peptide (GRP), is the most widely studied. GRP is the human homologue of the amphibian peptide, bombesin, and plays a role in embryonic development and adult repair of bronchial epithelia [3]. The GRP receptor, hereafter termed GRPR, is a G-protein-coupled receptor and member of the bombesin (BB) receptor family: BB1 receptor is activated by neuromedin B (NMB); BB2 (also called GRPR) is activated by GRP; the BB3 receptor shares only 50% homology with BB1 and BB2 and is an orphan receptor with an unidentified endogenous ligand.

The expression of bombesin-related peptides and the BB receptors by SCLC cell lines and primary tumours have been widely studied for the past three decades [3–6]. GRP and NMB secretion by SCLC is known to cause an autocrine growth loop that drives tumour growth. Therefore, a number of experimental therapeutics or imaging agents targeted at the GRP-GRPR interaction, including anti-GRP antibodies and GRPR peptide antagonists have been developed. These agents have demonstrated antitumour responses in a number of preclinical models of pancreatic cancer [7] and imaging of breast [8], pancreatic [9] and glioma [10] tumours.

The use of GRPR antagonists is motivated by their inability to cause downstream cell growth effects, but is counter-balanced by a greatly reduced rate of receptor internalisation. Nonetheless, a number of reports have illustrated that GRP/bombesin-based antagonists display superior in vivo targeting capacity cf. agonist peptides [11]. It is therefore reasonable to exploit the enhanced targeting capacity of the antagonist peptides for enhanced targeting of SCLC. Indeed, we postulate that high-affinity binding of antagonist peptides to SCLC cell surface GRPR would be expected to increase the local accumulation of the liposomes in the cell surface, thus increasing the probability of drug accumulation in the target cells, without activating GRPR signalling. For example, by increasing the fraction of liposomes that are membrane-bound through GRPR binding cf. non-targeted liposomes, which exchange rapidly between the free and cell surface-bound state, would indirectly increase the relative intracellular accumulation. Access to the intracellular compartment would be achieved by constitutive plasma membrane endocytosis (e.g., pinocytosis), resulting in membrane internalisation, endosome formation and trafficking through the endo-lysosomal system.

Nanomedicines to improve cancer therapy have been widely studied and have resulted in a number of approved therapies such as Doxil^®^ in the 1990s and the recent approval of Onivyde^®^ [12]. In the lung cancer field, cisplatin formulated as a pegylated liposomal formulation (Lipoplatin^®^) has delivered comparable antitumour response against non-small cell lung cancer tumours with reduced side effects when delivered in combination with paclitaxel compared to when cisplatin and paclitaxel solutions are used in combination [13]. In preclinical studies, improved therapeutic responses have been achieved by adopting an active targeting approach. Typically, this involves the incorporation of a surface-bound moiety that selectively binds to a cognate receptor/protein on the tumour cell surface, leading to accumulation of nanocarriers in the tumour. A diversity of targeting ligands has been explored, including antibodies, proteins, peptides and aptamers. Targeted nanoparticles such as HER2-targeted MM-302 [14], transferrin receptor-targeted CALAA-01 [15], and prostate-specific membrane antigen (PSMA)-targeted BIND-014 [16] have reached the clinic but detailed information about the clinical advantage of using targeted platforms is still forthcoming. In this study we explored whether surface engraftment of a GRPR antagonist peptide could be used to target GRPR expressing lung cancer cells for the purposes of enhanced liposome delivery to lung cancer.

## Results and Discussion

### GRPR as a target in lung cancer

**Figure 1:**
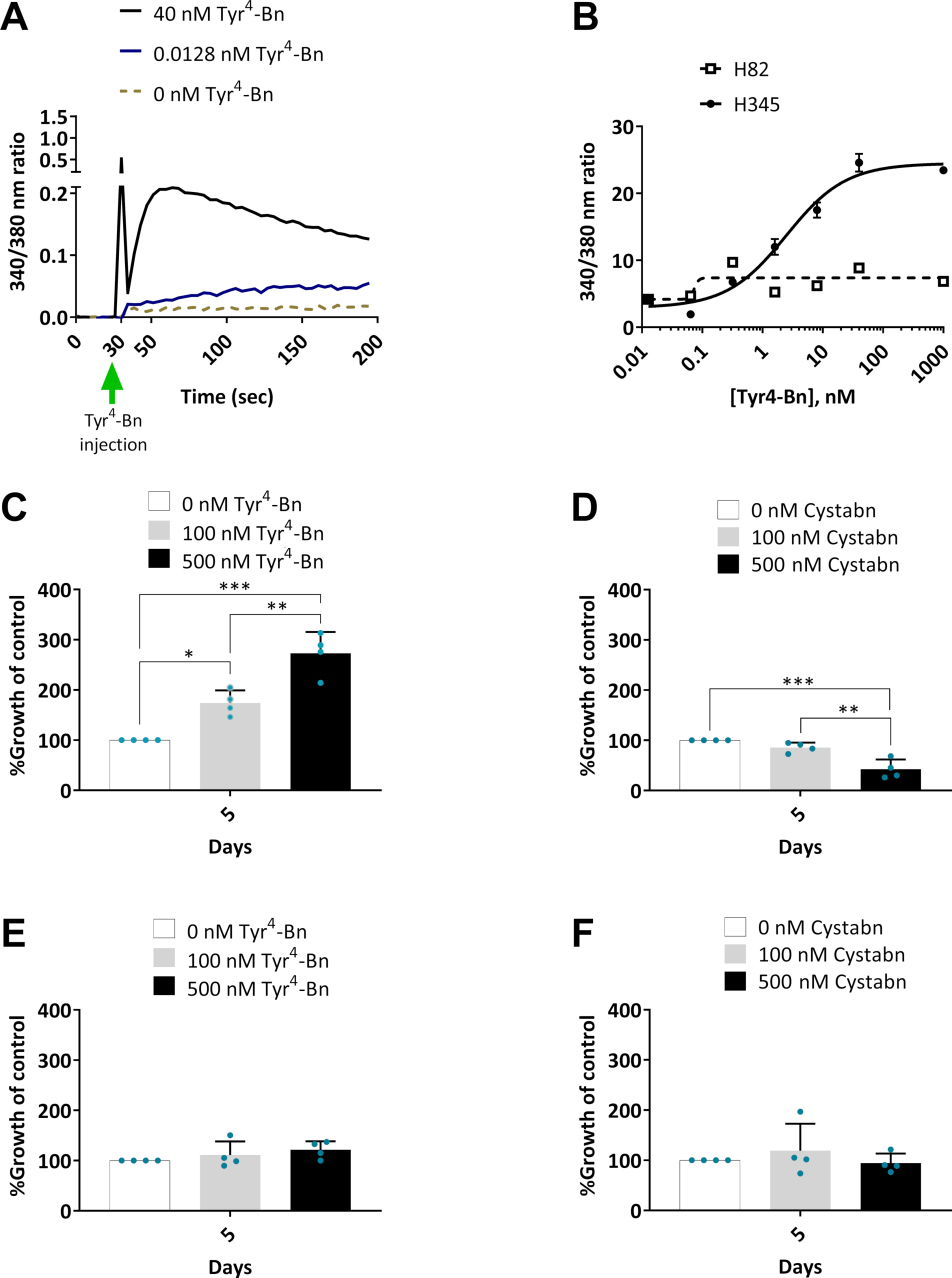
GRPR functionality in lung cancer cells. (A) Exemplar fluorescence trace from H345 cells loaded with Ca^2+^ reporter dye, Fura-2 AM before injection of GRPR agonist, Tyr^4^-Bn. Baseline fluorescence was monitored for 30 s before peptide injection. (B) Escalating concentrations of Tyr^4^-Bn were injected into H345 or H82 cells and the Fura-2 AM fluorescence emission monitored over 200 s. Peak Fura-2 ratios at 340/380 nm were plotted against agonist concentration. Data are mean ± SD. H345 cells were cultured over 5 days in selenite-insulin-transferrin (SIT) serum-free medium in the presence of escalating concentrations of Tyr^4^-Bn (C) or cystabn (D) before cell growth quantification by MTS assay. Similarly, (E, F) show MTS growth data for H82 cells in the presence of Tyr^4^-Bn (E) or cystabn (F). Error bars are SD.

The functionality of GRPR in SCLC cells was confirmed by Fura-2 studies in which NCI-H345 or NCI-H82 SCLC cell models were exposed to a dose-range of the canonical GRPR agonist peptide, Tyr^4^-Bn. A dose-dependent increase in intracellular calcium release was observed for NCI-H345 but not the GRPR deficient SCLC line, NCI-H82 (Figure 1a,b), as previously reported [17–18]. The physiological role of GRPR includes the stimulation of a mitogenic response after receptor internalisation [19]. SCLC growth as a result of stimulation by nanoparticle targeting ligands is highly undesirable. We therefore sought to explore the use of GRPR antagonist peptides on the liposomal carriers. Here, we exploited a peptide derived from the work of Mansi et al. who showed that the radiolabelled antagonist peptide, ^111^In-DO3A-CH_2_CO-G-aminobenzoyl-ᴅ-Phe-Q-W-A-V-G-H-Sta-Leu-NH_2_ increased by 3.5-fold the relative tumour accumulation of radiolabel compared to the agonist peptide, ^111^In-DO3A-CH_2_CO-G-aminobenzoyl-Q-W-A-V-G-H-L-M-NH_2_ [20–21]. This report reinforced the concept that the use of GRPR antagonist peptides was strongly preferable to the use of agonist peptides which have the potential to fuel tumour growth. The use of antagonists in preference to agonists is not, however, accepted by all. Different groups, using different agonist/antagonist peptide pairs as well as different imaging probes and visualisation approaches, have reached different conclusions about which approach should be used [22–23]. Based upon preferable in vitro and in vivo properties reported by others we based our work on the statine (Sta)-based antagonist peptide, ᴅ-Phe-Gln-Trp-Ala-Val-Gly-His-Sta-Leu-NH_2_.

The published peptide structure was modified to bear an N terminal ʟ-cysteine residue, which enables subsequent attachment to a functionalised lipid carrier. The peptide, hereafter termed cystabn, was prepared by fluorenylmethoxycarbonyl (Fmoc) solid-phase peptide synthesis and characterised by HPLC and mass spectrometry (data not shown). To confirm the persistent functionality of the peptide after cysteine addition, NCI-H345 cells were exposed to escalating concentration of Tyr^4^-Bn and cystabn in serum-free conditions. As expected, Tyr^4^-Bn resulted in a scalable increase (*p* < 0.05) in cell number as judged by MTS assay (Figure 1c). In contrast, exposure to cystabn, reduced cell proliferation over a 5 day period (Figure 1d), thus confirming that the addition of an N terminal cysteine did not interfere with GRPR binding. Exposure of NCI-H82 cells to the Tyr^4^-Bn agonist (Figure 1e) and cystabn antagonist peptide (Figure 1f) caused no change in cell growth.

These cell proliferation studies were performed in serum-free conditions in line with previous studies [24–25] for two principle reasons. Firstly, the removal of serum from the culture medium depletes bovine bombesin-like peptides that could otherwise stimulate cell growth. Secondly, the presence of serum proteases could rapidly reduce the integrity of the peptides, as demonstrated for other bombesin-related peptides that displayed mouse and human serum half-lives measured in the tens of hours [26]. The specificity of Tyr^4^-Bn and cystabn activity was confirmed in GRPR depleted NCI-H82 cells. In contrast to results from NCI-H345 cells, the addition of Tyr^4^-Bn to NCI-H82 caused no noticeable increase in cell proliferation and cystabn effected no reduction in proliferation. This demonstrated that cystabn functionally targeted GRPR expressing cells in a specific manner – a key feature of a targeting ligand to enable high tumour localisation.

### Formulation of a GRP-targeted liposomal carrier

**Figure 2:**
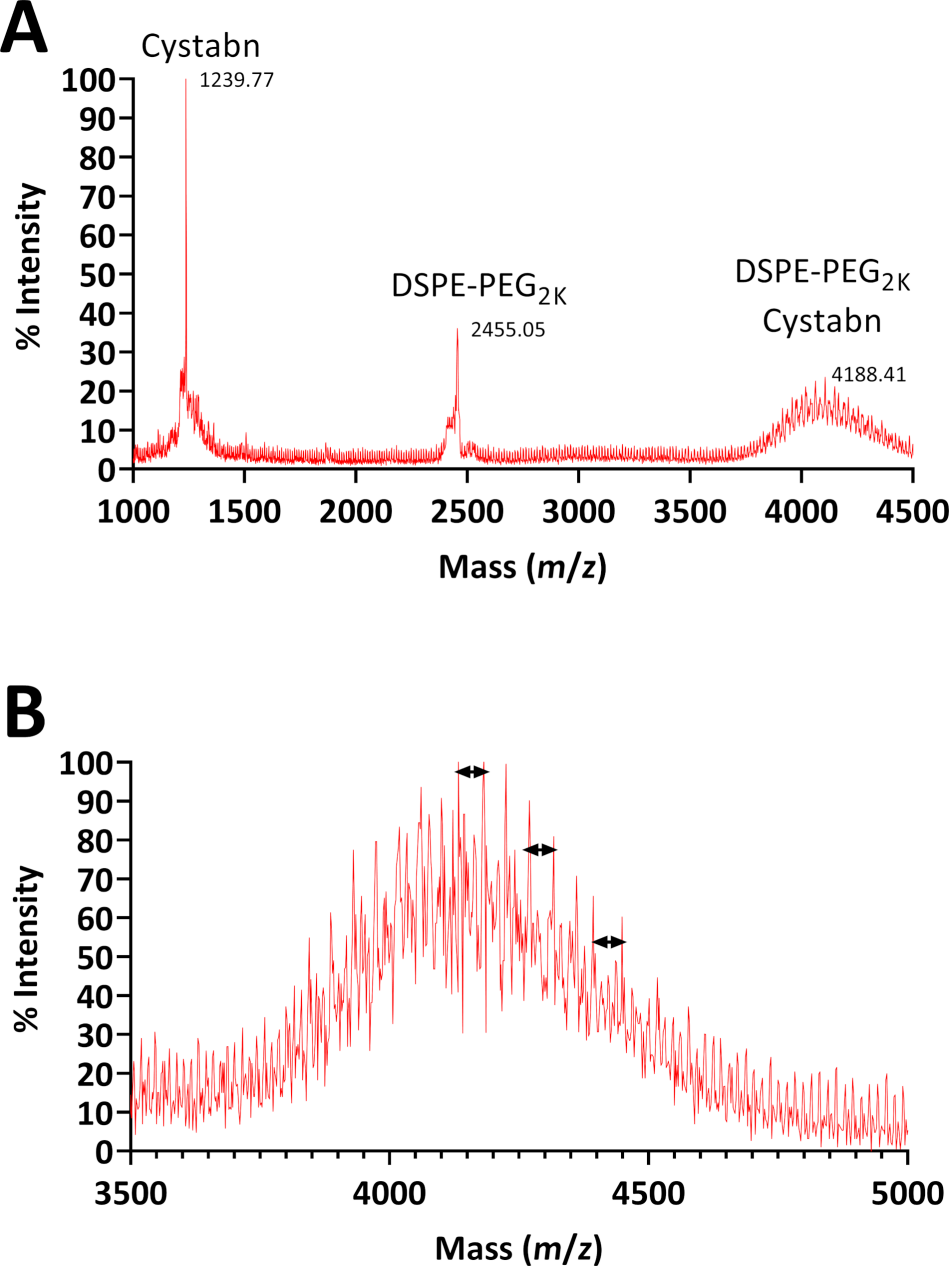
Mass spectrometry characterisation of cystabn-lipid conjugate. MALDI–TOF mass spectra of crude DSPE-PEG_2000_-cystabn conjugate (A) and purified, post-dialysis, DSPE-PEG_2000_-cystabn conjugate (B).

Having validated GRPR as a functional target on the surface of SCLC cells we next prepared a targeted liposomal carrier. The presence of a single thiol group in the cystabn peptide affords facile functionalisation of DSPE-PEG_2000_-maleimide thorough thiol coupling chemistry. Successful conjugation of the peptide was confirmed by MALDI–TOF mass spectrometry (Figure 2a,b). The most abundant mass peak of DSPE-PEG_2000_-cystabn (calculated mass: 4137.41 Da, assuming perfect monodispersity of the PEG_2000_ group) was observed at 4188 Da (Figure 2a) and was separated from surrounding peaks by 44 Da, which represents the mass of a single ethylene glycol unit (Figure 2b).

Liposomal formulations were developed to incorporate DSPE-PEG_2000_-cystabn or control DOPE-PEG_2000_ (see below Table 2, in section Materials). Liposomes without targeting cystabn peptide (control-lipo) contained 5 mol % DOPE-PEG_2000_, whereas targeted formulations (target-lipo) were loaded with 3 mol % of targeting DSPE-PEG_2000_-cystabn conjugate with the total mass of PEG-lipid made up to 5% with DOPE-PEG_2000_. The formulations were prepared using the thin-film technique to yield small and monodisperse vesicles as judged by dynamic light scattering (DLS) analysis (Table 1).

**Table 1:** Colloidal properties of control- and target-lipo formulations.^a^

	control-lipo	target-lipo (3 mol % cystabn conjugate)

Z-Ave (d.nm)	101 ± 2.1	90 ± 0.6
PDI	0.058 ± 0.007	0.050 ± 0.020
zeta potential (mV)	−1.64 ± 2.13	−2.15 ± 0.2

^a^Data shown are mean ± SD, *n* = 3 independent experiments.

**Figure 3:**
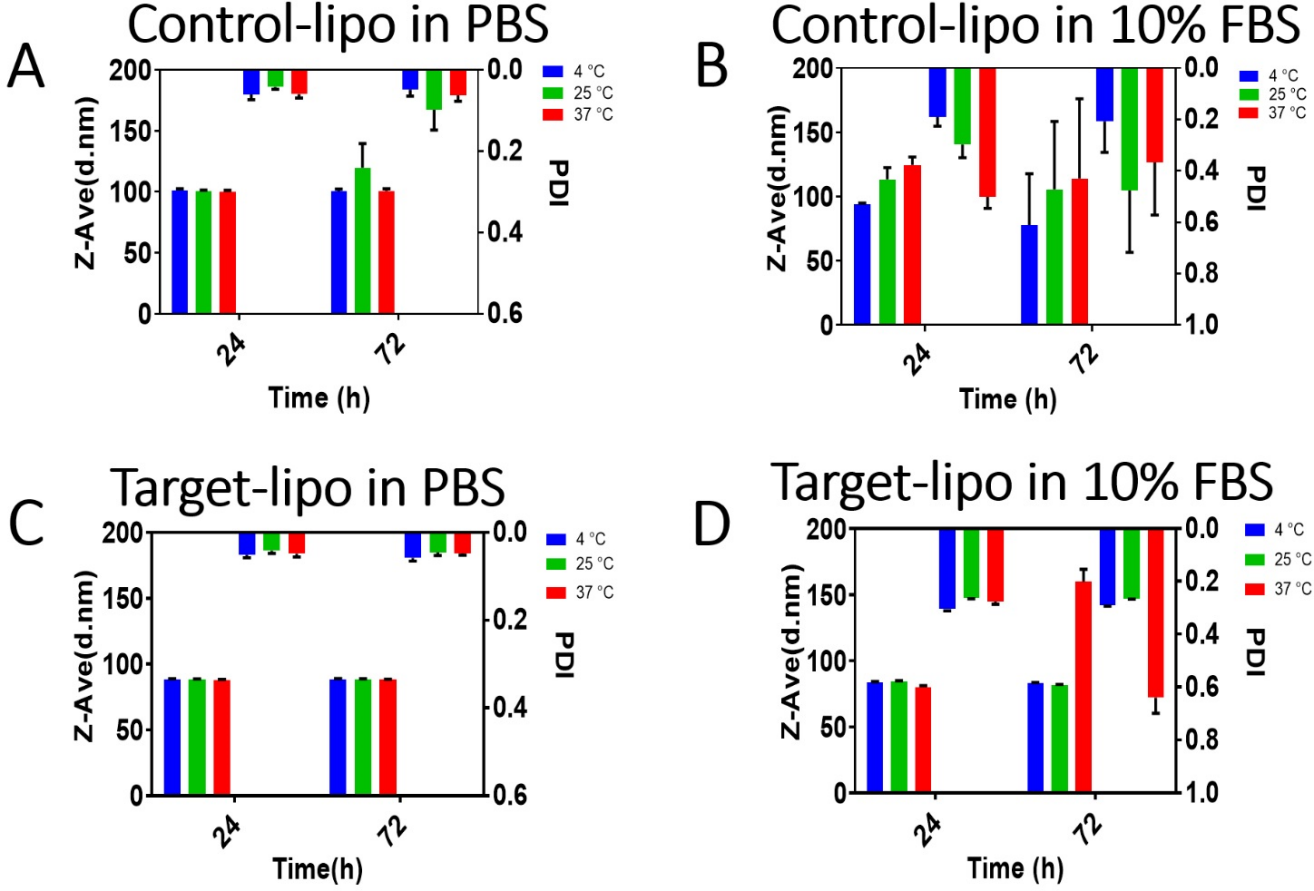
Colloidal stability of liposomes. Control and target liposomes were exposed to PBS (A,C) or 10% FBS in PBS (B,D) at three temperatures for 24/72 h then analysed by DLS. Data shown are mean ± SD, *n* = 3.

The colloidal properties of both liposomal formulations were highly similar in terms of size, polydispersity and zeta potential and consistent with those reported for other pegylated liposomes by others [27]. The vesicles were colloidally stable in PBS over 72 h at temperatures of 4, 25 and 37 °C with no significant changes in size, PDI or zeta potential observed (Figure 3a,b). It was noted that the diameter of both liposome formulations was larger than the 50 nm pore diameter of the terminal extrusion membrane. This is likely due to the deformation of the vesicles under pressure during extrusion and subsequent expansion after emergence from the pore.

Commercial realisation of targeted nanomedicines is contingent upon the development of platforms that are sufficiently resistant to aggregation in body fluids such as blood. The stability of control and targeted liposomes was examined after exposure to 10% heat-inactivated FBS for 24 or 72 h at different temperatures (Figure 3c,d). There were noticeable increased in liposome size and PDI as a function of both incubation time and temperature. For example, the control-lipo vesicles increased to 125 nm diameter after 24 or 72 h incubation at 37 °C. We noted a transition from a unimodal size distribution of liposomes in PBS buffer to a multimodal size distribution after extended incubation with 10% FBS. This caused sharp increases in the PDI of both formulations with increases in size and polydispersity (PDI) being greater for the target-lipo vesicles.

The emergence of particle populations with different sizes is not unexpected considering that FBS represents a complex, concentrated cocktail of polydisperse proteins of different sizes [27]. Indeed, the surface properties of various nanoparticles have been shown to change dramatically in the presence of plasma or serum [28] with the establishment of an adsorbed protein corona around the nanoparticle. It is now widely accepted that the particle protein corona presents a new biomolecular interface that underpins the dynamic interactions of nanosystems and their biological targets. The presentation of a high affinity GRPR antagonist peptide on the liposomal surface is expected to maintain liposomal cell-binding affinity by virtue of its high affinity for the receptor. Although, steric blockade of the targeting motif is possible due to the dynamic exchange events association with the evolution of the hard and soft corona, even on pegylated liposomes.

### Cell binding and uptake of GRPR-targeted liposomes

The targeting capacity of cystabn-decorated liposomes was examined using two different approaches. To examine this, we exploited a GRPR overexpression construct transfected into the widely used, adherent non-SCLC cell line A549. This model grows more rapidly than SCLC cells and its adherent phenotype allows for easier manipulation. First, quantification of cell-uptake was judged by flow cytometry analysis of A549-GRPR cells exposed to fluorescently tagged target-lipo (FL-Target-lipo) or tagged control-lipo (FL-Control-lipo) formulations. Preliminary studies using FL-Target-lipo including 1 mol % targeting lipid showed marginal increases in relative cellular accumulation (data not shown). To overcome this, the density of targeting lipid was increased to 3 mol %. To examine for active internalisation and intracellular accumulation of liposomes at the endocytosis permissive temperature of 37 °C, we subtracted the median fluorescence intensity (MFI) attributable to cell-surface adsorption of liposomes at 4 °C, to yield a “normalised cell MFI” at each time point and for each formulation.

**Figure 4:**
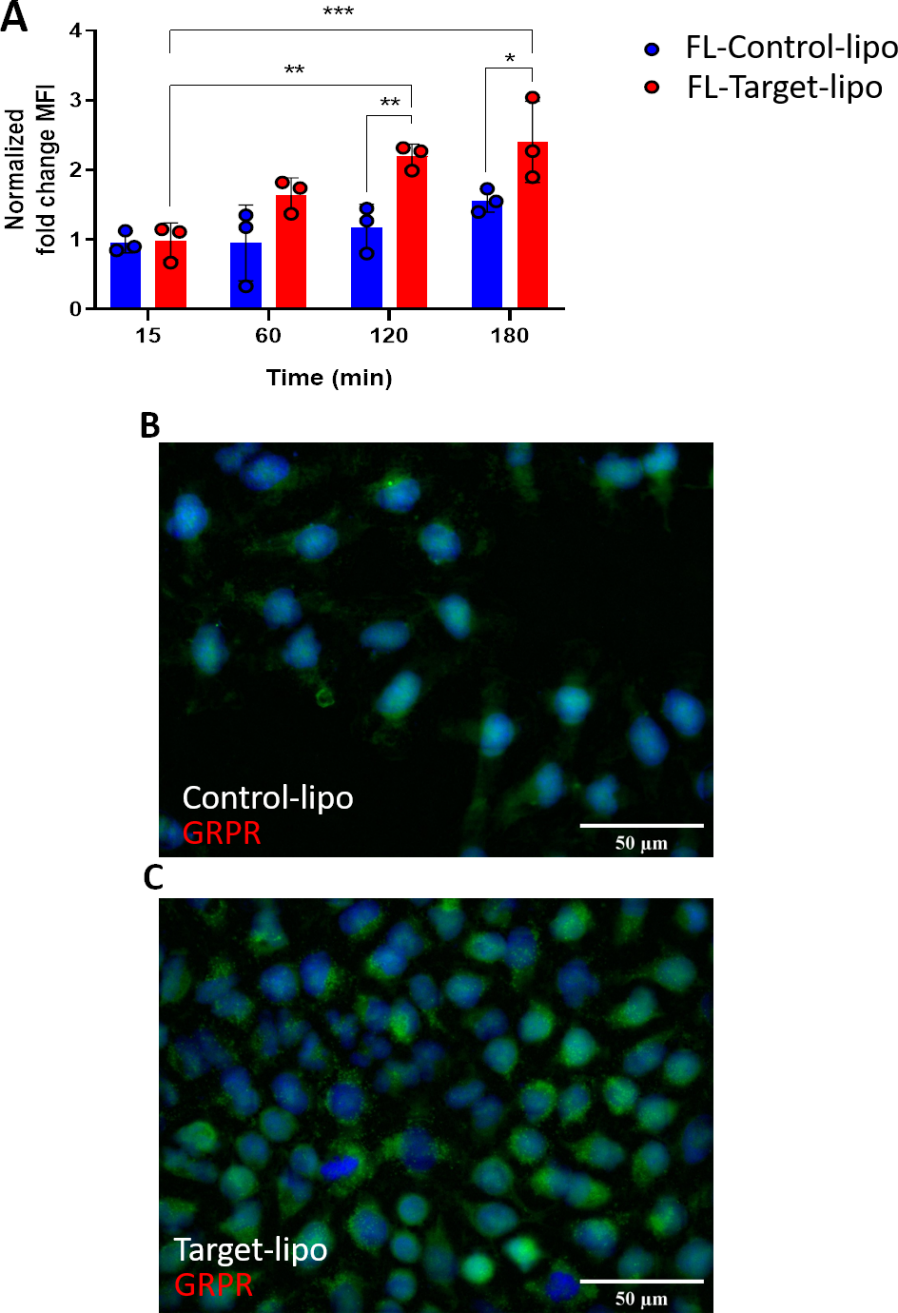
GRPR targeting with cystabn increases cell accumulation of liposomes. (A) A549-GRPR cells were exposed to 1 μg/mL of control or target liposomes for 15–180 min before washing and analysis of liposomal 1,2-dihexadecanoyl-*sn*-glycero-3-phosphoethanolamine (DHPE)-fluorescein intensity by flow cytometry. Fold-change median fluorescence intensity (MFI) was determined by correcting for the background MFI from cells exposed for matched time periods on ice. Data shown are mean ± SD, *n* = 3. A549-GRPR cells were exposed to 1 µg/mL of control (B) or target (C) liposomes (green) for 5 min, washed, fixed and nuclei labelled with Hoechst (blue).

For non-targeted FL-Control-lipo, there was a modest increase in normalised fluorescence over time (blue bars, Figure 4a). In contrast, the fluorescence of FL-Target-lipo increased over time (red bars, Figure 4a). Drawing comparison between the control and targeted liposome groups, it was clear to see that at 15 min, fluorescence was no greater in cells treated with FL-Target-lipo compared to FL-Control-lipo. However, at 60 min and beyond cell fluorescence was greater (*p* < 0.05) in cells treated with target-lipo.

We next confirmed the flow cytometric results using fluorescence microscopy. Following 5 min exposure to either control (Figure 4b) or target-liposomes (Figure 4c), A549-GRPR cells displayed greater cellular fluorescence signals in the FL-Target-lipo group. Cells exposed to FL-Control-lipo displayed a diffuse cell membrane-like staining with few green fluorescent puncta. Whereas, the target-lipo exposed cells displayed many more fluorescent puncta as well as a widespread increase in cellular fluorescence. Our observations here indicate that GRPR targeting with cystabn peptide increases cell uptake of liposomes, most likely through receptor-mediated uptake.

Taken together the flow cytometry and microscopy data demonstrate that, using a fluorescently labelled model liposomal carrier, the relative increase in cell uptake afforded by cystabn functionalisation is modest but significant. To put our data into context, approximately two-fold enhanced delivery of an oligonucleotide-bombesin [3,7–14] conjugate was observed by Ming et al. [29]. Using a similar approach to ours, Accardo et al. studied liposomal delivery of doxorubicin into PC-3 prostate cells using a modified bombesin targeting peptide [30]. The authors showed a reduction in mouse PC-3 xenograft size compared to non-targeted doxorubicin liposomes and saline control, consistent with tumour accumulation of the delivery system. In summary, an increase in SCLC cellular accumulation of a liposomal drug cargo would be beneficial for therapy, especially considering the chemotherapeutic resistance profile that is often displayed by clinical SCLC [31].

Future studies on cystabn-targeted liposomal carriers will examine the uptake and trafficking of these nanocarriers, particularly with regards to their efficiency of carrying chemotherapeutic agents into the cell. Poor intracellular accumulation of nanocarriers can be improved through targeted and triggered drug release, for example through the incorporation of temperature-sensitive [32] or light-sensitive lipids [33]. These approaches have shown promise in enhancing the cellular/tumoural accumulation of chemotherapeutic agents in various models [34–35].

In spite of the enhanced cellular delivery shown here, the application of GRPR targeting nanosized delivery systems to different cancers should be considered carefully due to variability in GRPR expression across malignant diseases. Published reports, using techniques such as reverse-transcriptase polymerase chain reaction (RT-PCR) and immunostaining, have shown that between 63–100% of prostate tumours are GRPR positive, while SCLC tumours are more heterogeneous, with 29–85% of tumours expressing GRPR [36]. This demonstrates that GRPR expression is not a universal marker of SCLC or any other tumour and that future development of GRPR-targeted therapeutics would require patient stratification according to the expression status.

## Conclusion

This report demonstrates that the functionalisation of liposomes with a GRPR antagonist peptide is sufficient to promote the accumulation of liposomes within GRPR expressing cells such as SCLC. Increased internalisation of drug-loaded GRPR targeted liposomes in treatment-resistant tumours such as SCLC could offer improved therapeutic outcomes.

## Experimental

### Materials

Fmoc-protected amino acids, piperazine, HCTU, HOBt, DMF were all from AGTC (Hessle, UK). Rink Amide MBHA resin was from Novabiochem (UK). HPLC solvents and all cell culture media were from Sigma-Aldrich (Poole, UK). Lipids were from Avanti Polar Lipids (USA). Sources of other exceptional items are mentioned in the text.

### Calcium release assay

NCI-H345 and NCI-H82 cells were loaded with 2 µM Fura-2AM (Thermo Fisher, UK) in Hank’s Balanced Salt Solution (HBSS) loading buffer containing sulfinpyrazone at 37 °C for 40–60 minutes. Following loading, the buffer was replaced with HBSS and allowed to warm to 37 °C in a Flexstation 3 microplate reader (Molecular Devices). Fluorescence of Fura-2 was recorded using dual excitation (340 and 380 nm) and emission 520 nm. Agonist was automatically injected using the Flexstation compound plate and tips at a specific time point. PMT settings were medium and 3 reads performed per well. Data is represented as 340/380 ratio using zero baseline for normalisation.

### Cell proliferation assay

The proliferation of NCI-H345 and NCI-H82 cells in the presence or absence of Tyr^4^-Bn and cystabn was studied in selenium–insulin–transferrin (SIT) medium comprising 30 nM sodium selenite, 5 µg/mL human insulin, 10 μg/mL human transferrin and 2 mM ʟ-glutamine in RPMI-1640 media. The cells were seeded in 96 well plates overnight at a density of 30,000 cells per well. The cells were then treated with 100 or 500 nM Tyr^4^-Bn or cystabn and for 5 days with peptide replenishment at day 3. Cell proliferation was determined using the CellTiter 96^®^ AQueous One assay (Promega).

### Peptide synthesis

Cystabn (Cys-ᴅ-Phe-Gln-Trp-Ala-Val-Gly-His-Sta-Leu-NH_2_) was synthesized on the Rink Amide MBHA resin using Fmoc chemistry. After initial deprotection of the resin with 2 × 5 min 5% piperazine in DMF, each coupling step involved addition to 1 equiv of resin, Fmoc-protected amino acid, HCTU, HOBt and DIPEA (4:4:4:8 equiv) in 1.5 mL DMF. The mixture was incubated for 30 min at RT with occasional gentle agitation then washed with DMF (×3). This process was repeated to increase the peptide yield. Successful Fmoc removal and coupling of amino acids was confirmed with the Kaiser test. After successful coupling and Fmoc deprotection of the N-terminal amino acid, the resin was washed with DMF (3 × 5 mL, 1 min), then DCM (3 × 5 mL, 5 min) and the resin dried under nitrogen then stored in vacuo for 3–5 h. Simultaneous side chain deprotection and peptide cleavage from the resin was achieved using TFA/TIPS/EDT/H_2_O (94:2:2:2), performed at RT for 4 h. The peptide was precipitated in diethyl ether overnight at −20 °C. Precipitated peptide was harvested by centrifugation (3000 rpm, 10 min, 4 °C). Peptide precipitate was washed (×3 cold diethyl ether) then dissolved in 10% aqueous acetic acid and lyophilised to yield a white powder.

### Peptide-PEG-lipid conjugate synthesis

One equivalent of DSPE-PEG_2000_-maleimide (6.8 μmol in 4 mL chloroform) was added to two equivalents of cystabn (13.3 μmol in 2 mL methanol) and stirred for 24 h at RT under nitrogen gas. After confirmation of successful conjugation by MALDI–TOF mass spectrometry analysis the solvent was evaporated and the reaction mixture re-dissolved in milli-Q water. Unreacted peptide was removed by dialysis against milli-Q water at RT for three days (the water changed every 2 h then left overnight) using 2 kDa cut-off benzoylated dialysis tubing (SpectroPor, Spectrum Labs, New Brunswick, USA). The purified conjugate was lyophilised to a dry white powder.

### Characterisation of peptides and conjugates

#### HPLC

Samples were analysed using a gradient elution method using mobile phase A (H_2_O + 0.1% TFA) and B solution (acetonitrile + 0.1% TFA) on a Perkin Elmer HPLC system comprising of binary solvent pump, autosampler, UV–vis detector and Peltier column oven. Mobile phases were membrane degassed using Millipore vacuum filtration with a 0.2 µm filter. The gradient profile was 0–5 min 5% B, 5–25 min 5–95% B and 5 min 5% B. Peptide samples (≈1 mg/mL in milli-Q water) were eluted on a Phenomenex Luna^®^ C18 (2) LC Column (5 µm, 100 Å, 150 × 4.6 mm, Phenomenex, UK). Conjugate analysis was performed on a Hypersil™ BDS C8 LC Column (3 µm, 130 Å, 150 × 4.6 m, Thermo Scientific, UK). Samples of 10 µL were injected and elution monitored at 280 nm.

#### MALDI–TOF

Peptide samples (2 mg/mL in 1:1 acetonitrile: milli-Q water + 0.1% TFA) were mixed with an equal volume of a saturated solution of α-cyanohydroxycinnamic acid (Sigma) and 1 µL spotted twice onto the same well of a clean MALDI sample plate. Peptide-PEG_2000_-lipid conjugates were dissolved in chloroform at 2 mg/mL and a 1:1 mixture prepared with saturated methanolic solution of universal MALDI matrix (Sigma-Aldrich, UK). Samples were analysed using linear ion detection mode on a SHIMADZU Axima-CFR MALDI–TOF.

### Liposomal formulation

Lipids for each liposomal formulation were dissolved in chloroform and mixed in a round-bottomed flask (see Table 2, below). The concentration of lipid-PEG-peptide conjugate solutions in chloroform were determined by UV spectroscopy using the molar extinction coefficient for the peptide tryptophan residue (5560 AU/mmol/mL). A thin film was produced by slow evaporation of the solvent under vacuum followed by one hour under high vacuum to remove solvent traces. The film was hydrated with PBS to a final lipid concentration of 10 mg/mL then heated to 55 °C and vortexed extensively to produce MLVs. Five cycles of freeze-thawing (dry ice-acetone followed by heating to 55 °C) were performed to produce reduce the lamellarity of the vesicles. Finally, lipid suspensions were extruded (21×) through polycarbonate membranes of 200 nm, 100 nm and 50 nm pore sizes to produce a narrow size distribution of LUVs.

**Table 2:** Formulation components of liposomes reported in this study.

formulation name	components

control-lipo	DOPC/cholesterol/DOPE-PEG2000 (57:38:5 mol %)
target-lipo	DOPC/cholesterol/DOPE-PEG2000/DSPE-PEG2000-cystabn (57:38:2:3 mol %)
FL-Control-lipo	DOPC/cholesterol/DOPE-PEG2000/DHPE-fluorescein (56:38:5:1 mol %)
FL-Target-lipo	DOPC/cholesterol/DOPE-PEG2000/DSPE-PEG2000-cystabn/DHPE-fluorescein (56:38:2:3:1 mol %)

#### Characterization of liposomal formulations

Liposomes were characterized for size and zeta potential using Zetasizer Nano ZS. For size measurements the liposomal suspension was diluted 1:10 with PBS. For zeta potential measurements, the liposomal suspension was diluted 1:10 with PBS and transferred to a clean folded capillary cell (Malvern, DTS1070).

#### Liposomal stability in PBS and 10% serum

An aliquot of the liposomal suspension was diluted 1:10 with either PBS or 10% FBS in PBS and transferred to individual microcentrifuge tubes. Samples were incubated at 4, 25 or 37 °C. After 0, 24 and 72 hours the samples were transferred to cuvettes and measured for size and PDI as described above.

### Cell culture and transfection

The adherent, non-small cell cancer cell line A549 was stably transfected with a plasmid encoding HA epitope-tagged human GRP receptor (3xHA-GRPR pcDNA3.1+ plasmid #GRPR00TN00, UMR cDNA Resource Center, USA) using FuGene 6 and selected using 750 μg/mL G418 over three weeks. Cells were subsequently maintained in 100 μg/mL G418.

#### Flow cytometry

A549-GRPR cells were washed with warm PBS and blocked for 5 h in 0.2% BSA in RPMI-1640 medium. The cells were dissociated using Versene and aliquoted at a concentration of 10^6^ cells per mL in phenol-red free SFM. The samples were incubated at 37 or 4 °C for 5 min before treating with Control-lipo or Target-lipo (1 µg/mL total lipids concentration) for 15–180 min. After incubation at 37 °C, cells were transferred onto ice and washed using 500 µL phenol red-free SFM. Samples were analysed on a Beckman Coulter CytoFlex flow cytometer using exited using 488 nm laser and the emitted wavelength acquired using 585/42 bandpass filter. After doublet exclusion, 10^4^ events/sample were acquired in the gated population, and analysed using CytExpert software (v2.3, Beckman Coulter, USA).

#### Fluorescence microscopy

Cells were seeded onto 16 mm coverslips, and incubated for 24 h. Cells were washed (×3) with PBS for 5 min at RT and treated with 1 µg/mL (total lipid) of fluorescein-labelled control or target liposomes for 5 min at 37 °C. Coverslips were washed (×3) with PBS, fixed with 4% PFA (10 min at RT) and residual PFA quenched by 50 mM NH_4_Cl (15 min, RT). The cells washed with PBS (×3) for 5 min, permeabilised with 0.2% Triton X-100 (10 min). Nuclei were stained with Hoechst 33258, 1 µg/mL in PBST) then mounted on glass slides using Prolong Gold mounting medium (Invitrogen). The cells were viewed under a widefield microscope (Zeiss AxioPlan 2ie) and images taken using Axiovision software and analysed using Fiji software.
